# The healing journey: incorporating psychological and spiritual dimensions into the care of cancer patients

**DOI:** 10.3747/co.v15i0.269

**Published:** 2008-08

**Authors:** A.J. Cunningham

**Keywords:** Mind, body, spirit, research, healing, suffering, stress, coping, psychological

## Abstract

Research on the factors that promote healing of the body through mind and spirit is at a very early stage. Reliance on experimental designs seems premature; we need much more exploratory research to identify relevant variables and useful therapeutic approaches before applying to them the same methods used to evaluate drugs. The Healing Journey is a program that has been in operation since 1982 at the Princess Margaret Hospital, Toronto, Ontario. Observational data collection, followed by qualitative analysis has demonstrated benefits for many cancer patients.

